# Safety, feasibility, and early efficacy of the water-specific 1940-nm laser wavelength for ablation of saphenous incompetence

**DOI:** 10.1016/j.jvscit.2023.101125

**Published:** 2023-02-14

**Authors:** H. Hong Keo, Cassandra Somma, Christian Regli, Daniel Staub, Nicolas Diehm, Juliane Lindenberg, Roman Gaehwiler, Heiko Uthoff

**Affiliations:** a Vascular Institute Central Switzerland, Aarau, Switzerland; b Department of Angiology, University Hospital and University of Basel, Basel, Switzerland; c Gefässpraxis am See – Lakeside Vascular Center, Lucerne, Switzerland

**Keywords:** 1940-nm Diode laser, DVT, EHIT, Occlusion, Pain, Saphenous vein, Varicose vein

## Abstract

**Objective:**

The aim of the present study was to evaluate the safety, feasibility, and early efficacy of saphenous vein ablation using a water-specific 1940-nm diode laser wavelength using low linear endovenous energy density.

**Methods:**

We retrospectively analyzed a series of patients who had undergone endovenous laser ablation (EVLA) between July 2020 and October 2021 from the multicenter, prospectively maintained VEINOVA (vein occlusion with various techniques) registry. EVLA was performed using a water-specific 1940-nm radial laser fiber. In the same session, all insufficient tributaries were treated by phlebectomy or sclerotherapy. Tumescent anesthesia was injected into the perivenous space. The vein diameter, energy delivered, and linear endovenous density were reviewed at baseline. The incidence of venous thromboembolism, endovenous heat-induced thrombosis (EHIT), burns, phlebitis, paresthesia, and occlusion were reviewed at 2 days and 6 weeks of follow-up. We used descriptive statistics to describe the results.

**Results:**

Overall, 229 patients were identified. Of the 229 patients, 34 were excluded because of treatment of recurrent varicose veins at a previously operated site (residual or neovascularization). Finally, 108 patients with varicose veins and 87 with recurrent varicose veins (new varicose veins in an untreated area) due to disease progression were included in the present analysis. A total of 256 native saphenous veins (163 great saphenous veins, 53 small saphenous veins, and 40 accessory saphenous veins) in 224 legs had undergone EVLA. The mean patient age was 58.3 ± 16.5 years. Of the 195 patients, 134 (68.7%) were women and 61 (31.3%) were men. Nearly one half of the patients had a history of saphenous vein surgery (44.6%). The CEAP (clinical, etiology, anatomy, pathophysiology) class was C2 in 31 legs (13.8%), C3 in 108 (48.2%), C4a to C4c in 72 (32.1%), and C5 or C6 in 13 legs (5.8%). The treatment length was 34.8 ± 18.3 cm. The mean diameter was 5.0 ± 1.2 mm. The average linear endovenous density was 34.8 ± 9.2 J/cm. Concomitant miniphlebectomy was performed in 163 patients (83.6%) and concomitant sclerotherapy in 35 patients (18%). At 2 days and 6 weeks of follow-up, the occlusion rate of the treated truncal veins was 99.6% and 99.6%, respectively, with only one truncal vein (0.4%) with partial recanalization at 2 days and 6 weeks of follow-up. No cases of proximal deep vein thrombosis, pulmonary embolism, or EHIT had occurred at follow-up. Only one patient (0.5%) had developed calf deep vein thrombosis at 6 weeks of follow-up. The incidence of postoperative ecchymosis was rare (1.5%) and had resolved at 6 weeks of follow-up.

**Conclusions:**

EVLA of incompetent saphenous veins using the water-specific 1940-nm diode laser wavelength is feasible and appears to be safe and efficient with a high occlusion rate, minimal side effects, and a zero rate of EHIT.

Varicose veins arising from chronic venous disorders are highly prevalent and contribute to significant pain, debility, and quality of life reduction.^1^ Endovenous thermal ablation has been considered the reference standard for the treatment of saphenous vein incompetence since 2011.^2^, ^3^, ^4^ The reasons for the widespread use of endovenous thermal ablation include the faster recovery time, improvement in quality of life, and lower complication rates compared with surgical high ligation and stripping.^2^ Among the endovenous thermal ablation procedures, endovenous laser ablation (EVLA) is the method most often used worldwide at present.^5^ EVLA uses thermal energy to induce shrinkage of the vein wall and occlusion of the vein. When first introduced, a diode laser with a shorter wavelength of 810, 840, 940, or 980 nm was used. These wavelengths had a high absorption coefficient of oxyhemoglobin.^6^, ^7^, ^8^ The laser wavelengths used at present have a high absorption coefficient for water and are in the range of 1064, 1320, 1470, 1500, and 1940 nm.^8^ The higher the absorption of laser energy in water, the greater the energy absorbed in the vein wall.^5^^,^^9^ The use of a 1470-nm laser wavelength has provided excellent results, mostly at a linear endovenous energy density (LEED) of 60 to 90 J/cm.^10^, ^11^, ^12^ Thus, the focus has been on the side effects occurring after EVLA. The most common side effects seen with all laser types have been bruising, localized pain, induration, and discomfort along the treated vein.^13^ Four studies comparing different wavelengths (810 nm vs 980 nm,^14^ 810 nm vs 1320 nm,^15^ 940 nm vs 1320 nm,^16^ and 980 nm vs 1500 nm^17^) demonstrated that laser devices with a longer wavelength produced fewer side effects at a comparable LEED compared with those with a shorter wavelength. The use of a water-specific laser with longer wavelengths and a radial fiber tip resulted in significantly less pain and less ecchymosis during recovery.^10^^,^^18^^,^^19^ Mathematical remodeling also revealed that the water-specific longer laser wavelength results in better absorption of the laser energy by the vein wall and, thus, requires less energy to achieve wall damage and, consequently, reduces the rate of side effects.^20^ However, clinical data on the 1940-nm laser wavelength are limited.

The aim of the present study was to evaluate the safety, feasibility, and early efficacy of saphenous vein ablation using the water-specific 1940-nm diode laser wavelength and a low LEED.

## Methods

We performed a retrospective observational study using data from the ongoing multicenter VEINOVA (vein occlusion with various techniques) registry. The medical records of all consecutive patients who had undergone truncal saphenous vein treatment with EVLA using the 1940-nm diode laser wavelength at two centers between July 2020 and October 2021 were reviewed in accordance with the venous reporting standard guidelines.^21^ Our report was prepared in compliance with the STROBE (strengthening the reporting of observational studies in epidemiology) checklist.^22^ In accordance with the legal obligations in Switzerland, before the patients had made decisions concerning their treatment, all had received detailed written and verbal information about the proposed technique, the benefits and risks, and the alternative treatment options. Before we performed the procedure, all the patients had signed a written informed consent form and gave their consent for the investigator to use their data for scientific purposes. The study followed the principles outlined in the Declaration of Helsinki and was approved by the institutional review board (ethics committee Nordwest- und Zentralschweiz, project ID 2018-00,813).

The demographic data, vein characteristics, procedural data (including concomitant phlebectomy and sclerotherapy), and outcomes data (including ultrasound findings and complications) were assessed and extracted from the medical records. All the data were collected prospectively and entered into a database. Superficial venous insufficiency had been diagnosed for all patients from the duplex ultrasound findings. Saphenous vein incompetence was assessed with reflux in response to manual calf compression or the Valsalva maneuver with the patient standing. Reflux was defined as evidence of reverse flow of >500 ms in a vein segment.^23^

EVLA was performed by experienced vascular specialists with experience treating several hundred patients with EVLA using a 1470-nm diode laser and radiofrequency ablation. Bilateral treatment was allowed. Tumescent anesthesia was used in all cases of EVLA, which were performed as outpatient procedures. No sedation was routinely given. No limitation was placed on the vein diameter. Details of the procedure have been previously described.^24^

In brief, on the day of treatment, the location of the veins to be treated was mapped on the patient’s leg with the patient standing and using ultrasound guidance (Aplio a; Canon Medical System Europe, Zoetermeer, The Netherlands). Percutaneous cannulation of the great saphenous vein (GSV), small saphenous vein (SSV), or anterior accessory saphenous vein (AASV) was performed at the distal point of insufficiency under ultrasound guidance using the Seldinger technique. The access point was mostly infragenual for the GSV, mid-thigh for the AASV, and the distal calf for the SSV. A 16-gauge angiographic needle was used for vein puncture without the use of any separate introducer sheath or guide wire. After insertion of the laser fiber (400-μm iMS Diffuse Emission Fiber; iMS, Tutzing, Germany) through the sheath of the 16-gauge needle sheath, the fiber tip was advanced to the saphenofemoral junction (SFJ) or saphenopopliteal junction (SPJ) and then positioned 1 to 0.5 cm distal to the SFJ or SPJ with ultrasound guidance and connected to a 1940-nm radial diode laser device. The distance from the laser tip to the SFJ or SPJ was always measured using ultrasound to ensure a safe distance. Local tumescent anesthesia was prepared using 500 mL of 0.9% saline, 50 mL of 1% Rapidocaine (lindocaine hydrochloride monohydrate; Anesiva, Inc, South San Francisco, CA), and 5 mL of 8.4% sodium bicarbonate. Local tumescent anesthesia was then infiltrated into the perivenous space under ultrasound guidance using a motor pump. After tumescent anesthesia had been administered, the position of the laser tip was again verified, and the distance to the SFJ or SPJ was measured before activating the laser. Laser energy was then released at 3 W using a continuous mode, aiming for a LEED delivery of 20 to 40 J/cm. The pullback speed per 1 cm was set at 10 seconds and controlled by a ringtone, with the aim of a LEED at 30 J/cm. However, the definitive pullback speed was controlled at the operator’s discretion according to changes in the diameter, without exceeding a mean LEED of 40 J/cm, whenever possible. After EVLA, the refluxing tributaries were removed by phlebectomy or closed with sclerotherapy during the same procedure. After tumescent anesthesia placed alongside the tributaries, 1- to 3-mm incisions over the varicosities were performed, and the varicose tributaries were removed using a hook (Oesch; Salzmann AG, St Gallen, Switzerland). Concomitant foam sclerotherapy was performed alone or in addition to phlebectomy using ≤10 mL of 1% to 3% Aethoxysklerol (hydroxypolyethoxy-dodecane; Kreussler Pharma, Wiesbaden, Germany) mixed 1:4 with air.

After treatment, the legs were wrapped in sterile absorbent bandages and covered with a compressive cohesive bandage for those patients who had undergone concomitant phlebectomy. At follow-up at 2 days, the bandage was removed, and a duplex ultrasound scan was performed to evaluate for truncal occlusion and deep vein thrombosis (DVT), including endovenous heat-induced thrombosis (EHIT). The patients were asked whether they had experienced pulmonary embolism (PE) symptoms. If clinical suspicion for PE was present, computed tomography was performed. The patient was then instructed to wear a class 2 compression stocking during the day for 1 week. Compliance regarding the use of the stockings was not monitored. At 6 weeks of follow-up, all side effects were checked and recorded. The efficacy end point was the total occlusion rate of the treated truncal vein at 2 days and 6 weeks, and the main safety end point was the occurrence of EHIT, DVT, PE, or major bleeding at 2 days and 6 weeks of follow-up. The secondary safety end point was the occurrence of phlebitis, paresthesia, burns, infection, and minor bleeding. Ecchymosis or minor bleeding was defined as clinically apparent bleeding (ie, at least one episode of clinically apparent melena or hematemesis, spontaneous gingival bleeding, or epistaxis lasting for >5 minutes) and hemorrhagic wound complications (ie, excessive wound hematoma or wound hematoma leading to an unplanned consultation, hospitalization, or prolonged inability to work).

All the patients who had undergone EVLA routinely received thromboprophylaxis with a dose of 10 mg of rivaroxaban (Bayer AG, Zurich, Switzerland) once daily for 3 days. The first dose of the anticoagulant was usually administered at 3 hours postoperatively. The 3-day regimen of thromboprophylaxis was arbitrary and based on the belief that after 3 days the patient would have fully recovered and be fully mobilized. Routine mobilization was encouraged for the postoperative period without any limitations.

### Statistical analysis

Descriptive statistics with categorical data are presented as frequencies and percentages and continuous data as the mean ± standard deviation. Data analyses were performed using Stata statistical software, version 15, release 10 (StataCorp, College Station, TX).

## Results

A total of 229 patients who had undergone EVLA with the 1940-nm diode laser wavelength from July 2020 to October 2021 were identified in the ongoing VEINOVA registry. Of the 229 patients, 34 were excluded because these patients had undergone EVLA of recurrent varicose veins at a previously treated site (residual or neovascularization). Finally, 108 patients with varicose veins and 87 with recurrent varicose veins (new varicose veins in an untreated area) due to disease progression were included in the present analysis. A total of 29 patients had received bilateral treatment. The mean age of the total cohort was 58.3 ± 16.5 years. Of the 195 patients, 134 (68.7%) were women and 61 (31.3%) were men. Only seven patients (3.6%) had a history of venous thromboembolism, and three (1.5%) had thrombophilia. Of the 195 patients, 44.6% had a history of varicose vein surgery. Detailed patient characteristics are presented in Table I.

**Table I:** Baseline patient characteristics (N = 195)

Demographic characteristics	No. (%) or mean ± SD
Female sex	134 (68.7)
Age, years	58.3 ± 16.5
BMI, kg/cm^2^	25.9 ± 3.9
History of phlebitis	13 (6.7)
History of VTE	7 (3.6)
History of varicose vein surgery	87 (44.6)
Thrombophilia	3 (1.5)
CEAP class per leg (n = 224)	
C2	31 (13.8)
C3	108 (48.2)
C4a	62 (27.7)
C4b	3 (1.3)
C4c	7 (3.1)
C5	6 (2.7)
C6	7 (3.1)

*BMI,* Body mass index; *CEAP,* clinical, etiology, anatomy, pathophysiology; *SD,* standard deviation; *VTE,* venous thromboembolism.

CEAP (clinical, etiology, anatomy, pathophysiology) class was C2 in 31 legs (13.8%). The most prevalent CEAP class was C3 in 108 legs (48.2%), followed by C4a with pigmentation (n = 62; 27.7%). Cases of C4b, C4c, C5, and C6 were rare (Table I).

A total of 256 truncal varicose veins (163 GSVs, 53 SSVs, and 40 AASVs) in 224 legs were treated using the 1940-nm diode laser wavelength. The mean treatment length for the total cohort was 34.8 ± 18.3 cm. The mean diameter was 5.0 ± 1.2 mm for the total cohort. The average LEED administered for treating the truncal vein was 34.8 J/cm. Concomitant phlebectomy was performed in the same session for 83.6% of patients. Details of the procedure variables for the treated veins are given in Table II.

**Table II:** Lesion characteristics and procedural data per truncal vein (N = 256)

Characteristic	No. (%) or mean ± SD
GSV	163 (63.7)
SSV	53 (16.8)
ASV	40 (15.6)
Energy applied, J	1199 ± 675
LEED, J/cm	34.8 ± 9.2
Vein length, cm	34.8 ± 18.3
Total diameter, cm	5.0 ± 1.2
GSV diameter, cm	5.3 ± 1.2
SSV diameter, cm	4.5 ± 0.8
ASV diameter, cm	4.5 ± 1.1
Concomitant sclerotherapy (N = 195)	35 (18.0)
Concomitant phlebectomy (N = 195)	163 (83.6)

*ASV,* Accessory saphenous vein; *GSV,* great saphenous vein; *LEED,* linear endovenous energy density; *SD,* standard deviation; *SSV,* small saphenous vein.

At 2 days of follow-up, serious side effects, such as DVT, PE, EHIT, or major bleeding, had not occurred. At 6 weeks of follow-up, one case of calf DVT had developed. The incidence of postoperative phlebitis, paresthesia, burns, and minor bleeding was very infrequent. The observed complete occlusion rate of the treated truncal vein was 99.6% (255 of 256) at 2 days and 6 weeks follow-up. One case of partial recanalization (0.4%) was documented at 2 days and 6 weeks of follow-up. Details of the outcome variables are presented in Table III.

**Table III:** Safety and efficacy data

Outcome variable	Follow-up	
	At 2 days	At 6 weeks
Follow-up days	2.1 ± 0.7	40.4 ± 9.3
Total occlusion of truncal vein	255 (99.6)	255 (99.6)
Partial recanalization of truncal vein	1 (0.4)	1 (0.4)
Complete recanalization of truncal vein	0 (0)	0 (0)
DVT	0 (0)	1 (0.5)
PE	0 (0)	0 (0)
EHIT class I	0 (0)	0 (0)
EHIT class ≥II	0 (0)	0 (0)
VTE + EHIT class ≥II	0 (0)	1 (0.5)
Phlebitis	1 (0.5)	1 (0.5)
Paresthesia	3 (1.5)	6 (3.1)
Burns	1 (0.5)	1 (0.5)
Infection	0 (0)	0 (0)
Minor bleeding	3 (1.5)	3 (1.5)
Major bleeding	0 (0)	0 (0)

*DVT,* Deep vein thrombosis; *EHIT,* endovenous heat-induced thrombosis; *PE,* pulmonary embolism; *VTE,* venous thromboembolism.

Data presented as mean ± standard deviation or number (%).

## Discussion

In the present study, we assessed the safety, feasibility, and early efficacy of saphenous vein ablation using a water-specific 1940-nm diode laser wavelength and a low LEED. Early in the use of EVLA, shorter wavelength were used. However, as it became evident that absorption of laser energy varies at different chromophores and that the occurrence of postoperative complications is dependent on the wavelength, a trend began toward using a water-specific longer laser wavelength instead of a shorter wavelength.^25^ Thus, the present study has provided important data on the safety, feasibility, and early efficacy of varicose vein ablation using a 1940-nm diode laser wavelength. We found demonstrated that the early efficacy was high using an average LEED of 35 J/cm. In another study, histologic and immunohistochemical examination of the GSV after EVLA with the 1940-nm laser wavelength and LEED values of 50 vs 100 J/cm revealed excessive destruction to the intima and media layer that had caused high-grade thermal damage with a high LEED of 100 J/cm.^26^ Thus, a lower LEED with the 1940-nm laser wavelength was suggested to achieve effective occlusion with less high-grade thermal damage to the intima and media and to prevent damage to the adventitia and perivenous tissue, supporting the low LEED used in the present study. Other studies have also shown that water-specific laser wavelengths are significantly more powerful than hemoglobin-specific laser wavelengths and that the direct transfer of energy to the vein enables a lower LEED to achieve adequate vein wall destruction. Thus, despite less energy delivered to the vein wall, significant shrinkage occurred for the veins treated with a water-specific longer laser wavelength.^12^^,^^27^ This findings compare well with our results, showing a 99.6% occlusion rate.

Successful EVLA depends on effective transfer of the laser energy to the vein wall itself. The light energy must be absorbed and converted into heat and result in endothelial denaturation and vein wall shrinkage. LEED is the term used to quantify the amount of energy delivered per centimeter (J/cm) and depends on the wattage used for the laser but not the wavelength. Longer wavelengths have a high absorption coefficient for water and, thus, will be absorbed by the water molecules in the endothelial cells of the vein wall.^28^ Using low power (less wattage) and low LEED, the results of thermal damage to the perivenous tissue will also decrease, and a faster recovery can be expected with less postprocedural pain and morbidity.

Overall, the incidence of side effects such as burns and ecchymosis was very low in the present study. Insoo^5^ demonstrated a consistently low pain score after EVLA using a 1940-nm laser wavelength. From our personal experience using a 1470-nm laser and bare fiber at 10 W and mean LEED of 60 to 90 J/cm to treat incompetent saphenous veins, we believe that the experience of postprocedural pain was greater with the 1470-nm laser than with the 1940-nm laser wavelength. However, we did not monitor the patients' pain scores.

An interesting finding from the present study was the zero rate of EHIT. EHIT describes thrombotic extension from the treated truncal vein up to the junction or into the deep venous system. The zero rate of EHIT could have been because the water-specific laser wavelength transfers the energy directly to the endothelium of the vein wall, leading to effective shrinkage of the vein and, thus, less thrombus propagation into the deep system.^12^ One might argue that the zero EHIT rate had resulted from the small study population. However, in other studies with a similarly small study population that used a shorter laser wavelength (810-nm wavelength), the incidence of EHIT was still high, reported at 6.4% of 234 laser procedures performed.^29^ Whether the longer laser wavelength or lower LEED was responsible for the zero EHIT rate could not be evaluated statistically owing to the zero EHIT event rate and no comparison with a higher LEED. In addition, the zero rate of EHIT could have resulted from the administration of thromboprophylaxis. To clarify this hypothesis, the same study would have to be repeated without prophylactic anticoagulation. It has been shown in a previous multivariate analysis that the laser wavelength was an independent risk factor for EHIT but that the energy applied was not.^27^ Thus, the use of a longer laser wavelength might lead to a lower EHIT incidence. Some possible risk factors, such as a large GSV diameter (>8.5 mm), a history of venous thromboembolic disease, and male sex, have been associated with EHIT.^30^ However, the evidence has been inconsistent. Also, when ablation is started >2.5 cm distal to the SFJ or SPJ, a trend toward a decreased incidence of EHIT was found. However, the quality of the evidence is low (grade 2; level of evidence, C).^30^ In addition, these studies had used a laser wavelength of <1940 nm. Precision is presumed to be greater with the 1940-nm laser wavelength because the energy delivered is more selectively absorbed by the vein wall; thus, fewer complications should occur.

The present study had several limitations. First, our study was limited by its retrospective design, inherent to observational studies, and the lack of a comparison group treated with a lower wavelength. Second, we did not obtain a venous severity score during the evaluations; therefore, we could not include this information in our analysis. Finally, hypercoagulable risk factors such as the Caprini score were not assessed routinely as a part of our workup; thus, the effects of these factors on our results could not be determined. Despite these limitations, the present study has provided valuable information on the safety and early efficacy using the water-specific 1940-nm laser wavelength for ablation of incompetent saphenous veins in an ambulatory setting, given the limited data currently available. Our study has provided a clear protocol of low power and low LEED for efficient varicose vein ablation. Another advantage of the present study was the multicenter aspects of the observational design with prospective data collection. It would be interesting to compare the postoperative morbidity of EVLA with the 1470-nm and 1940-nm diode laser. Until such data and longer follow-up become available, the present study has provided valuable information on the practical aspects using a 1940-nm laser wavelength for varicose vein ablation.

## Conclusions

We found that EVLA of incompetent saphenous veins using a 1940-nm diode laser wavelength is feasible and appears to be safe and effective with a power at 3 W and average LEED of 35 J/cm. Furthermore, the occlusion rate was high with a low incidence of side effects and zero rate of EHIT for the patients who had routinely received a prophylactic dose of anticoagulation for 3 days after the procedure. A larger registry and longer follow-up comparing the 1940-nm and 1470-nm diode laser wavelength is clearly warranted to validate our findings.
